# Intradermal hepatitis B vaccination with imiquimod pretreatment in dialysis patients: a cost-effectiveness analysis

**DOI:** 10.1186/s12962-025-00682-0

**Published:** 2025-12-02

**Authors:** Yingcheng Wang, Mingjun Rui, Joyce H. S. You

**Affiliations:** School of Pharmacy, Faculty of Medicine, The Chinese University of Hong Kong, Hong Kong SAR, China

**Keywords:** Hepatitis B vaccination, Intradermal administration, Imiquimod, Dialysis patients, Cost-effectiveness analysis

## Abstract

**Background:**

Dialysis patients are at high risk for hepatitis B virus (HBV) infection. Intramuscular administration of HBV vaccination has shown limited responsiveness and durability of seroprotection in dialysis patients. Intradermal (ID) HBV Sci-B-Vac vaccination with imiquimod (Toll-like receptor 7 agonist) pretreatment was reported to be safe and improve seroprotection. This study aimed to evaluate the cost-effectiveness of ID administration of HBV Sci-B-Vac with pre-treatment imiquimod cream in dialysis patients from the perspective of US healthcare providers.

**Methods:**

A lifetime Markov model was used to estimate outcomes in a hypothetical cohort of serologically negative dialysis patients with: (1) HBV Sci-B-Vac administered intradermally with pre-treatment imiquimod (IMQ) cream (IMQ + ID group), (2) HBV Sci-B-Vac by intradermal injection alone (ID group), and (3) HBV Sci-B-Vac by intramuscular injection alone (IM group). Main results included direct medical costs, quality-adjusted life-years (QALYs), and incremental cost-effectiveness ratio (ICER).

**Results:**

QALYs in the IMQ + ID group (2.9763) were the highest, followed by the ID group (2.9751) and the IM group (2.9740). The ID group (more costly and totals less QALYs versus IMQ + ID) was strongly dominated by the IMQ + ID group, and was eliminated from the cost-effectiveness analysis. The ICER of the IMQ + ID group versus the IM group (17,032 USD/QALY) was lower than the willingness-to-pay (WTP) threshold (50,000 USD/QALY) and remained lower than the WTP threshold in the one-way sensitivity analysis. The probabilities of IMQ + ID, IM, and ID groups to be cost-effective at a WTP of 50,000 USD/QALY were 85.06%, 14.86%, and 0.08%, respectively.

**Conclusion:**

ID administration of HBV Sci-B-Vac with pre-treatment IMQ cream in serologically negative dialysis patients was the cost-effective strategy.

**Supplementary Information:**

The online version contains supplementary material available at 10.1186/s12962-025-00682-0.

## Background

Hepatitis B virus (HBV) infection presents a substantial threat to global public health, with complications such as liver failure, cirrhosis, and hepatocellular carcinoma (HCC) [1, 2]. In the US, the estimated prevalence of chronic HBV infection was 1.59 million cases in 2019 [3]. Patients on maintenance dialysis belong to a high-risk group for HBV infection, as repeated exposure to blood products and transfusions significantly increases the susceptibility to HBV infection [4, 5]. The immunocompromised state associated with end-stage renal disease (ESRD) in dialysis patients further increases the risk of progression, if HBV infected, to chronic HBV infection and serious complications [6].

HBV vaccine is recommended for all age groups [7]. Intramuscular (IM) administration of conventional alum-adjuvanted HBV vaccine is recognized as effective and safe for the general population. The responsiveness and durability of HBV vaccination were nevertheless lower in dialysis patients than in healthy adults [8]. Interventions to improve vaccine responsiveness and durability in this at-risk population were examined, including booster doses, higher vaccine dosages, the use of different adjuvants, and intradermal (ID) vaccination [9–12].

The HBV Sci-B-Vac is a third-generation recombinant vaccine expressing three hepatitis B surface antigens (pre-S1, pre-S2, and S). The pre-S1 and pre-S2 regions offer enhanced immunogenicity, which could surmount non-responsiveness to the S antigen [13]. A recent double-blind, randomized controlled trial assessed the efficacy of ID HBV Sci-B-Vac vaccine with topical Toll-like receptor 7 (TLR7) agonist imiquimod (IMQ) pretreatment in dialysis patients [14]. IMQ cream plus ID vaccination (IMQ + ID) was compared against two vaccination options: placebo cream plus ID vaccine (AQ + ID), and placebo cream plus IM vaccine (AQ + IM). The IMQ + ID group achieved a seroprotection rate (SPR) of 96.9% at 52 weeks, significantly higher than those of AQ + ID (74.2%) and AQ + IM (48.4%) groups (*p* < 0.0001) [14]. Despite the reported improvement in vaccine responsiveness, the cost-effectiveness of ID HBV vaccination with IMQ pretreatment is yet to be examined to inform decision-makers regarding the allocation of healthcare resources. The objective of this study is to evaluate the cost-effectiveness of ID HBV vaccination with IMQ pretreatment for dialysis patients from the perspective of US healthcare providers.

## Methodology

### Model design

**Fig. 1:**
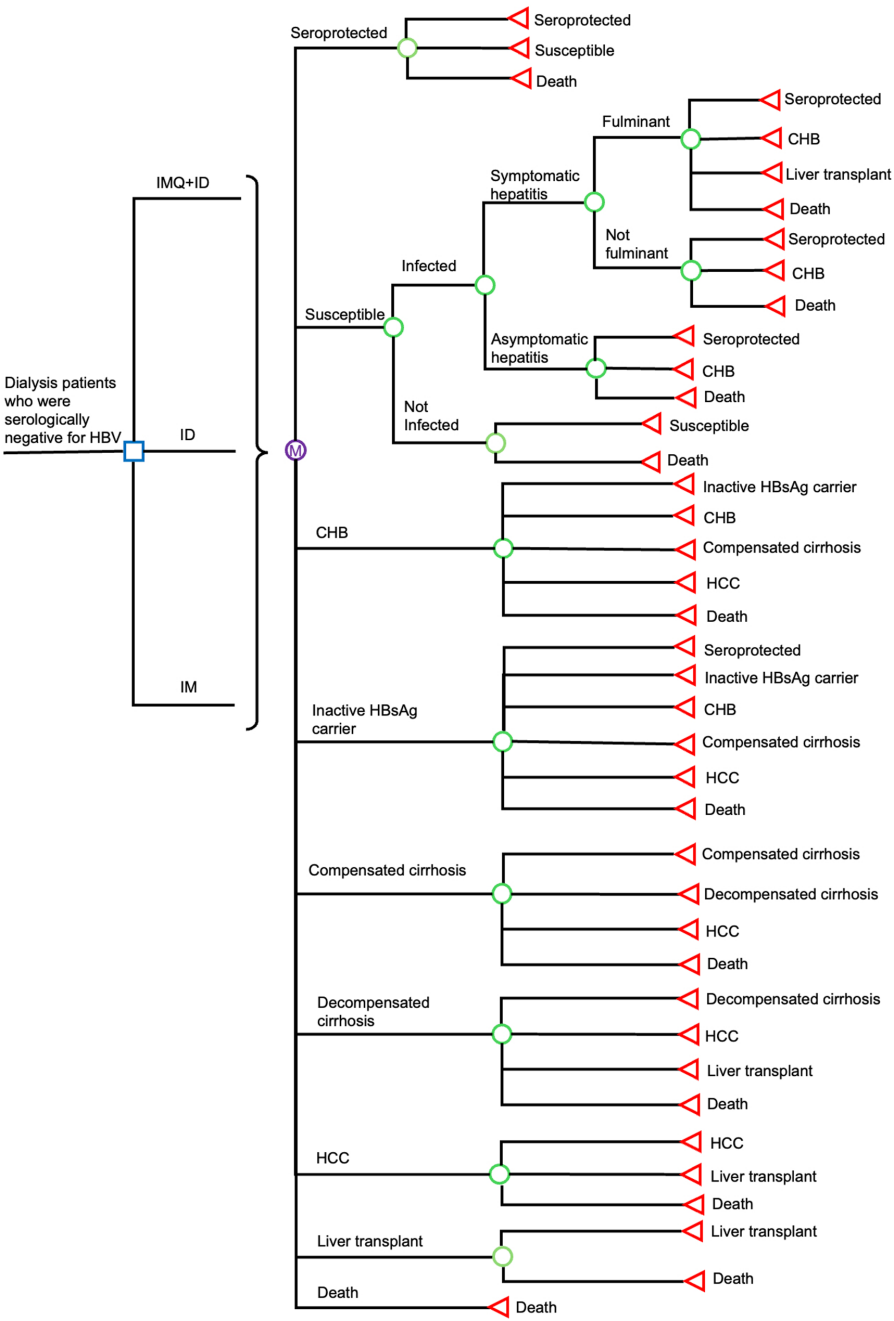
Simplified Markov model structure. IMQ + ID: intradermal HBV Sci-B-Vac with pre-treatment imiquimod cream; ID: intradermal HBV Sci-B-Vac; IM: intramuscular HBV Sci-B-Vac; HBV: hepatitis B virus; HBsAg: hepatitis B surface antigen; CHB: chronic hepatitis B; HCC: hepatocellular carcinoma. Square symbol: decision node; “M” symbol: Markov node; circle symbol: chance node; triangle symbol: end node

A Markov model (Fig. 1) was developed to simulate the expected costs and outcomes of three HBV vaccination strategies in three different hypothetical cohorts of serologically negative dialysis patients (on maintenance dialysis for at least 3 months). The HBV vaccination strategies were retrieved from the clinical study [14]: (1) ID HBV Sci-B-Vac with pre-treatment IMQ cream (IMQ + ID group), (2) ID HBV Sci-B-Vac (ID group), and (3) IM HBV Sci-B-Vac (IM group). Three different hypothetical cohorts of patients in three mutually exclusive health care programmes received the same four-dose regimen of HBV Sci-B-Vac (10 µg in 1 mL per dose), administered at 0, 4, 12, and 26 weeks. Those in the IMQ + ID group received topical IMQ cream (5%) before each ID HBV vaccination. Since the clinical trial observed only self-limiting adverse events with no medical resource implication, the model did not consider the impact of vaccine side effects [14]. The average age of patients starting dialysis (60 years) in the US was adopted in the present model [15]. The model applied a lifelong time horizon (with a yearly cycle) to capture the long-term costs and outcomes of the HBV vaccination in dialysis patients. The maximum life expectancy of ESRD patients at 60–64 years was reported to be 15.2 years, and a model timeframe of 15 years was therefore applied [16]. Main results included hepatitis B-related events, direct medical costs, quality-adjusted life-years (QALY) and incremental cost-effectiveness ratio (ICER).

All hypothetical patients entered the Markov model, after completion of 4-dose vaccination, at either “seroprotected” state (hepatitis B surface antibody (anti-HBs) titers ≥ 10 mIU/mL) or “susceptible” state [14]. The decision-analytical model structure is shown in Fig. 1, the Markov model included 9 health states (susceptible, seroprotected, chronic hepatitis B (CHB), inactive hepatitis B surface antigen (HBsAg) carrier, compensated cirrhosis, hepatocellular carcinoma, decompensated cirrhosis, liver transplant, death). The seroprotected patients might become susceptible, and susceptible patients might acquire acute HBV infection. Patients with acute HBV infection could be asymptomatic or symptomatic (with a risk of fulminant hepatitis and potential liver transplantation) and might progress to CHB. Patients with CHB might become inactive HBs-Ag carriers (tested positive for HBsAg for ≥ 6 months without symptoms or signs of liver disease, with negative hepatitis B e-antigen (HBeAg), normal liver enzyme levels, and serum HBV-DNA < 2000 IU/ml), or develop severe hepatitis B-associated complications (including compensated cirrhosis, decompensated cirrhosis, and HCC). Death might occur in all health states.

### Clinical inputs

All model inputs are listed in Table 1. The clinical inputs were identified from MEDLINE search over the period of year 2000 to year 2024. The starting year of literature search was dated from year 2000 to include clinical studies relevant to current clinical practice of dialysis care and HBV infection management. The selection criteria of clinical studies of HBV infection were: (1) Articles were written in English; (2) subjects were adults dialysis patients; and (3) outcomes of HBV infection or hepatitis B-related complications were reported. The detailed search strategies and the flowchart of the selection process are illustrated in the Supplementary Materials Table S1 and Figure S1. Epidemiology or disease burden studies in the US population, randomized clinical trials, and meta-analyses were the preferred sources.

**Table 1:** Model inputs

Parameters	Base-case value	Range for sensitivity analysis	Distribution	Reference
**Clinical inputs**				
Vaccination seroprotection rate				[14]
IMQ + ID	0.9690	0.7750-1.0000	Beta	
ID	0.7420	0.5940–0.8900	Beta	
IM	0.4840	0.3870–0.5810	Beta	
Yearly probability of acute HBV infection among susceptible dialysis patients	0.0017	0.0013–0.0020	Beta	[17, 18]
Proportion of symptomatic acute HBV infection	0.3000	0.1500–0.4500	Beta	[19]
Proportion of fulminant hepatitis among acute HBV infection	0.0830	0.0670-0.1000	Beta	[20]
Transition probability in dialysis patients (yearly)				
Seroprotected to Susceptible				[21]
IMQ + ID	0.0480	0.0350–0.0660	Beta	
ID	0.1280	0.1090–0.1500	Beta	
IM	0.3430	0.2990–0.3920	Beta	
Acute HBV infection to CHB	0.2725	0.2180–0.3270	Beta	[20]
Fulminant hepatitis to CHB	0.0710	0.0530–0.0890	Dirichlet	[17]
Fulminant hepatitis to Liver transplant	0.0010	0.0007–0.0020	Dirichlet	[17]
Fulminant hepatitis to Death	0.6700	0.5360–0.8040	Dirichlet	[17]
CHB to Inactive HBsAg carrier	0.1110	0.0710–0.2290	Dirichlet	[17]
CHB to Compensated cirrhosis	0.2000	0.1600–0.2400	Dirichlet	[20]
CHB to HCC	0.2000	0.1600–0.2400	Dirichlet	[20]
Inactive HBsAg carrier to Seroprotected	0.0054	0.0043–0.0065	Dirichlet	[22]
Inactive HBsAg carrier to CHB	0.0449	0.0359–0.0538	Dirichlet	[22]
Inactive HBsAg carrier to Compensated cirrhosis	0.0090	0.0072–0.0108	Dirichlet	[22]
Inactive HBsAg carrier to HCC	0.0024	0.0019–0.0028	Dirichlet	[22]
Compensated cirrhosis to Decompensated cirrhosis	0.1000	0.0800–0.1200	Dirichlet	[20]
Compensated cirrhosis to HCC	0.0440	0.0352–0.0528	Dirichlet	[20]
Compensated cirrhosis to Death	0.2326	0.1861–0.2791	Dirichlet	[23]
Decompensated cirrhosis to HCC	0.0630	0.0300–0.0700	Beta	[17]
Decompensated cirrhosis to Liver transplant	0.0000	0.0000-0.0170	Gamma	[17]
Decompensated cirrhosis to Death	0.4090	0.3280–0.4910	Beta	[23]
HCC to Liver transplant	0.0000	0.0000-0.0170	Gamma	[17]
HCC to Death	0.5120	0.4100–0.6150	Beta	[24]
Liver transplant to Death	0.4800	0.3840–0.5760	Beta	[20]
**Utility values**				
Dialysis patients	0.7000	0.6200–0.7800	Beta	[25]
Hepatitis B-related state utility in general population				
Symptomatic acute HBV infection	0.7000	0.6300–0.7700	Beta	[17]
Fulminant hepatitis	0.3700	0.3300–0.4100	Beta	[17]
Inactive HBsAg carrier	0.8500	0.7700–0.9400	Beta	[17]
CHB	0.6700	0.6400–0.7000	Beta	[26]
Compensated cirrhosis	0.6600	0.6100–0.7100	Beta	[26]
Decompensated cirrhosis	0.3700	0.3200–0.4200	Beta	[26]
HCC	0.4300	0.3600–0.5000	Beta	[26]
Liver transplant (year 1)	0.5700	0.5400–0.6000	Beta	[26]
Post Liver transplant	0.6400	0.5900–0.6900	Beta	[26]
**Cost data (USD)**				
HBV Sci-B-Vac (per dose)	34	27–41	Normal	[27]
ID needle (per unit)	10	8–12	Normal	[28]
Imiquimod cream (per sachet)	3	2–7	Normal	[29]
Vaccination administration (first dose)	20	18–27	Normal	[30]
Vaccination administration (addition dose)	15	13–19	Normal	[30]
Treatment of symptomatic acute HBV infection	10,239	3,594 − 16,883	Gamma	[17]
Treatment of fulminant hepatitis	28,026	22,421 − 33,631	Gamma	[31]
Liver transplant surgery	354,690	283,752 − 425,628	Gamma	[31]
State costs (annual)				
Inactive HBsAg carrier	183	146–220	Gamma	[30]
CHB	2,263	1,810-2,715	Gamma	[32]
Compensated cirrhosis	2,263	1,810-2,715	Gamma	[32]
Decompensated cirrhosis	19,970	15,976 − 23,964	Gamma	[32]
HCC	46,066	36,853 − 55,280	Gamma	[32]
Liver transplant	37,730	30,184 − 45,276	Gamma	[32]

HBV: hepatitis B virus; CHB: chronic hepatitis B; HCC: hepatocellular carcinoma; HBsAg: hepatitis B surface antigen

The annual probability of mortality of patients at the health states or transition events “susceptible”, “seroprotected”, “asymptomatic acute HBV infection”, “symptomatic acute HBV infection”, “inactive HBsAg carrier” and “CHB” was based on the age-adjusted all-course mortality in the US dialysis patients reported by the United States Renal Data System (USRDS) [24]

The percentages of dialysis patients who achieved seroprotection in the three vaccination strategies were retrieved from SPR findings reported in a randomized clinical trial (*n* = 94) at week 52 after vaccination. The SPRs were 96.9%, 74.2% and 48.4% in the IMQ + ID, ID and IM strategies, respectively [14]. The model addressed the assumption of waning immunity (transition probability from seroprotection to susceptibility) in dialysis patients based on findings of an observational study which traced the antibody in 392 first-time HBV vaccinees for 10 consecutive years [33]. This study stratified the vaccinees into low (10-100mIU/mL), high (100-1000mIU/mL), and very high (≥ 1000mIU/mL) response groups by the initial anti-HBs titer levels, and higher initial anti-HBs titers was associated with longer maintenance duration of anti-HBs [33]. The reported geometric mean concentration of anti-HBs in the IMQ + ID, ID, and IM groups aligned with those of the “very high”, “high”, and “low” response groups, respectively [14]. To estimate the decline rates of anti-HBs titers in IMQ + ID, ID, and IM groups, we applied several standard parametric survival models (exponential, Weibull, log-normal, log-logistic, and Gompertz distributions) to fit the Kaplan-Meier survival curves for the proportion of anti-HBs-positive persons in the “very high”, “high” and “low” response groups, correspondingly. The exponential distribution was selected based on superior visual fit and the lowest Akaike information criterion among the tested models. The presence of anti-HBs is universally recognized as immunity against HBV infection [21], and the decline rate was therefore used to estimate the transition probability from seroprotection to susceptibility.

The yearly probability of acute HBV infection in susceptible dialysis population (0.0017) was calculated using the yearly incidence rate of HBV infection (reported by the national surveillance of dialysis-associated diseases in the US [17, 18]) with the following formula [34]:$\:p=1-{exp}^{-rt},\:$where p = probability, t = time, and r = rate [35]. The proportion of symptomatic infections (30%) in acute HBV-infected cases was retrieved from a cost-effectiveness analysis comparing 10 µg and 5 µg doses of HBV vaccine in newborns [19]. The proportion of fulminant hepatitis (0.0830) among symptomatic acute HBV infection was adopted from the model input of a cost-effectiveness analysis study that estimated two HBV vaccines in the ESRD cohort [20]. The annual probability of progression from acute HBV infection to chronic hepatitis B (0.2725) was retrieved from a cost-effectiveness analysis for ESRD cohorts [20] (originated from the findings of a two-year follow-up study of 231 hemodialysis patients [36]).

The annual transition probabilities from inactive HBsAg carrier to other health states (seroprotected, CHB, compensated cirrhosis, and HCC) were estimated from a long-term follow-up (median period of 8.6 years) study in HBsAg carrier patients (*n* = 283) [22]. Annual transition probabilities of CHB to inactive HBsAg carrier, fulminant hepatitis (to CHB and liver transplant) and compensated cirrhosis (to decompensated cirrhosis and HCC) were adopted from cost-effectiveness analysis studies on HBV vaccines in cohorts with ESRD or chronic kidney disease (CKD) [17, 20]. Hepatitis B-related deaths included those from fulminant hepatitis, compensated and decompensated cirrhosis, HCC, and liver transplant states. The annual mortality probabilities of fulminant hepatitis and liver transplant state were adopted from the previous cost-effectiveness analyses on HBV vaccines in ESRD or CKD patients [17, 20]. For compensated cirrhosis, decompensated cirrhosis and HCC, the annual mortality probabilities were estimated from the survival analysis in population-based studies in hemodialysis patients diagnosed with cirrhosis or HCC [23, 24]. The annual probability of mortality of patients at the health states or transition events “susceptible”, “seroprotected”, “asymptomatic acute HBV infection”, “symptomatic acute HBV infection”, “inactive HBsAg carrier” and “CHB” was based on the age-adjusted all-course mortality in the US dialysis patients reported by the USRDS [37].

### Utility values

The expected QALY gains of each strategy were estimated from the utility values and duration spent in different health states. The utility of dialysis patients in specific hepatitis B-related health states was approximated using the utility of dialysis, adjusted with hepatitis B event-specific utility. The utility value of dialysis was retrieved from a systematic review and meta-analysis on health-related quality of life in late-stage CKD patients [25]. A multi-country study (*n* = 1,134) measured the utility values of six hepatitis B health states (chronic hepatitis B, compensated cirrhosis, decompensated cirrhosis, HCC, year-1 liver transplant, and post-liver transplant) using standard gamble through an interviewer-administered survey, and the present model adopted the utility values reported for the US subjects [26]. The utility value of symptomatic acute HBV infection, fulminant hepatitis and inactive HBsAg carrier were retrieved from a previous cost-effectiveness analysis study on HBV vaccines in CKD patients [17]. The QALYs were discounted to the current year with an annual discount rate of 3% [38].

### Costs data

The present analysis included direct medical costs from the perspective of the US healthcare provider. Cost items were vaccination costs and medical costs for hepatitis B-related events. In the search for costs of these resource items, the CDC vaccine price list, the physician fee schedule and diagnosis related groups (DRGs) (by the US Centers for Medicare & Medicaid Services) are reliable open sources and therefore used for estimating the costs and administration of vaccine, and cost of hepatitis B-related complications. The costs of HBV Sci-B-Vac, IMQ cream and ID needles were obtained from the CDC vaccine price list and online suppliers, respectively [33–28]. The costs of vaccine administration and serological testing were estimated from the physician fee schedule of the US Center for Medicaid and Medicare Services [30]. The costs of fulminant hepatitis treatment and liver transplant surgery were retrieved from the inpatient hospital data by DRG provided by the US Centers for Medicare & Medicaid Services [31]. The cost of symptomatic acute HBV infection treatments was adopted from a cost-effectiveness analysis of adult HBV vaccination [17]. The annual direct medical costs for health states of CHB, compensated cirrhosis, decompensated cirrhosis, HCC, and post-liver transplant were sourced from a retrospective study examining healthcare resource utilization and costs for CHB patients across insurance databases between 2004 and 2015 [32]. All costs were adjusted to 2023 US dollars [39] and were discounted at an annual rate of 3% [40].

### Base case and sensitivity analyses

All analyses were conducted in TreeAge Pro 2023 (TreeAge Software, Inc., Williamstown, MA, USA) and Microsoft Excel 365 (Microsoft Corporation, Redmond, WA, USA). A strategy was strongly dominated (and therefore eliminated from cost-effectiveness analysis) when it had a net loss of QALY at a higher cost (compared with at least one mutually exclusive option) [41]. A strategy was considered strongly dominant when it totals more QALYs and cost-saving versus at least one mutually exclusive option. After ranking health care programs in ascending order of cost, the ICER for the vaccination strategy—relative to the next least expensive option—was calculated by dividing the incremental costs by the incremental QALYs.

A strategy was considered cost-effective when the ICER was below the threshold of willingness-to-pay (WTP) threshold. A WTP threshold of 50,000 USD/QALY is a historically accepted benchmark in US cost-effectiveness analyses [42]. The present study therefore adopted this WTP threshold to ensure consistency and comparability across findings of health economics studies of HBV vaccination in the US.

The robustness of cost-effectiveness results was assessed by sensitivity analyses [41]. One-way sensitivity analysis examined the uncertainty of the baseline findings by varying one parameter at a time, according to its range, as showed in Table 1. Each parameter was varied by upper and lower limits, 95% confidence intervals (CIs), or plausible range using ± 20% of base-case value (when both upper/lower range and 95% CI were lacking) [41, 43]. The variations applied to each parameter to test its uncertainty are described in Supplementary Materials Table S2. Probabilistic sensitivity analysis was performed using 10,000-iteration Monte Carlo simulation, generated from simultaneously drawing random values of all the parameters which were given a theoretical probability distribution, as specified in Table 1. Details of distributions of all parameters are showed in Supplementary Materials Table S2. The incremental costs and incremental QALYs of the compared vaccination strategies were calculated for each of the 10,000 simulations, and presented as mean and 95% CIs. The 95% CI for incremental cost and QALYs were calculated using the mean and the standard error obtained from the 10,000-iteration Monte Carlo simulation. The probabilities of each strategy to be accepted as cost-effective over a range of WTP thresholds were presented in the cost-effectiveness acceptability curves [44, 45].

## Results

### Base-case analysis

The expected hepatitis B-related event rates of the three vaccination strategies are shown in Table 2. The event rates of the IMQ + ID group were lower than those of the IM and ID groups. The expected costs and QALYs per vaccinated patient are presented in Table 3. The QALYs in the IMQ + ID group (2.9763) were the highest, followed by the ID group (2.9751) and the IM group (2.9740). The ID group (more costly and had a net loss of QALYs versus IMQ + ID) was strongly dominated by the IMQ + ID group, and was eliminated from the cost-effectiveness analysis. When compared to the IM group, the IMQ + ID group gained 0.0023 QALYs with an ICER of 17,032 USD/QALY (less than the WTP threshold of 50,000 USD/QALY). The IMQ + ID group was therefore the cost-effective option in the base-case analysis.

**Table 2:** Expected number of acute HBV infections, fulminant hepatitis, CHB, HCC, liver transplant and hepatitis B-related death per 100,000 vaccinated Dialysis patients

Vaccination strategy	Acute infection	Fulminant hepatitis	CHB	HCC	Liver transplant	hepatitis B-related death
IM	802	20.0	214.5	71.6	0.020	129.7
ID	529	13.2	141.4	46.3	0.013	82.5
IMQ + ID	204	5.1	54.5	17.2	0.005	29.7

HBV: hepatitis B virus; CHB: chronic hepatitis B; HCC: hepatocellular carcinoma

**Table 3:** Expected costs and QALYs per vaccinated patient

Vaccination strategy	Costs (USD)	Incremental costs (USD)	QALYs	Incremental QALYs	ICER vs. the next less costly option
IM	287	-	2.9740	-	-
IMQ + ID	326	39	2.9763	0.0023	**17**,**032**
ID	335	9	2.9751	-0.0012	Strongly dominated by IMQ + ID

QALY: quality-adjusted life-year; ICER: incremental cost per QALY; Bold: ICER < Willingness-to-pay threshold (50,000 USD/QALY)

### Sensitivity analyses

**Fig. 2:**
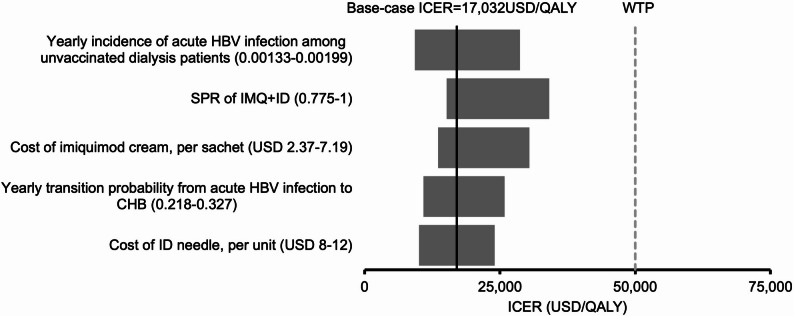
Tornado diagram of the variation of the ICER of the IMQ + ID group versus the IM group against the top five influential parameters identified in one-way sensitivity analysis. SPR: seroprotection rate; HBV: hepatitis B virus; CHB: chronic hepatitis B; HCC: hepatocellular carcinoma; WTP: willingness-to-pay; WTP = 50,000 USD/QALY

The ICER of IMQ + ID remained lower than the WTP threshold throughout the variation of all model inputs in the one-way sensitivity analysis. The top five influential factors on the ICER of the IMQ + ID versus the IM group are shown in a tornado diagram (Fig. 2): yearly incidence of acute HBV infection among unvaccinated dialysis patients, SPR of IMQ + ID, cost of IMQ cream, yearly transition probability from acute HBV infection to CHB, and cost of ID needle.

**Fig. 3:**
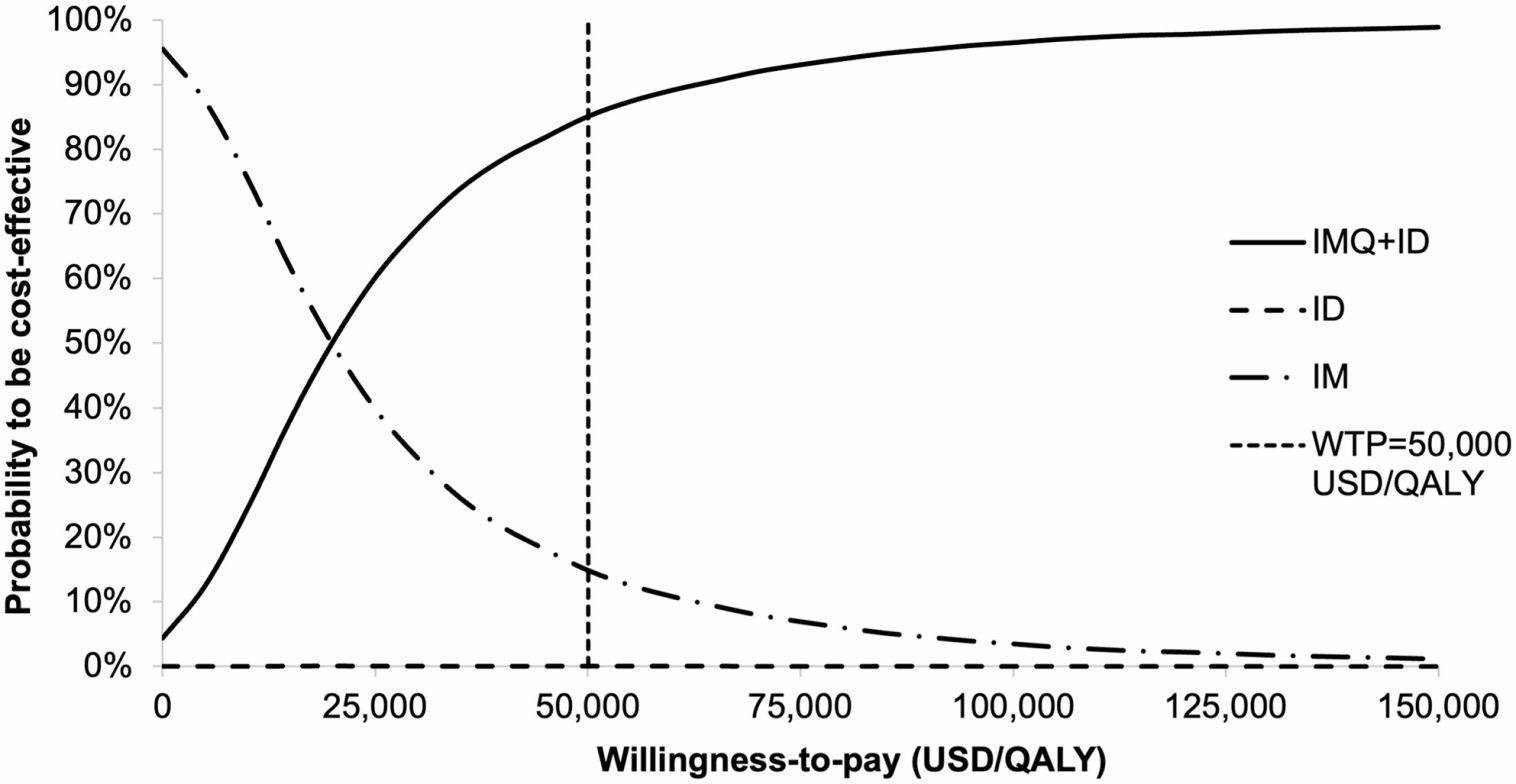
Variation in the probability of each vaccination strategy to be cost-effective against willingness-to-pay. QALY: quality-adjusted life year; WTP: willingness-to-pay

In the probabilistic sensitivity analysis, the mean QALYs gained by IMQ + ID group was 0.0020 QALYs (95% CI 0.002016–0.002049; *p* < 0.01) with a mean incremental cost of USD39.35 (95% CI 38.91–39.80; *p* < 0.01) when compared to the IM group. The ICERs of IMQ + ID versus IM were lower than the WTP threshold in 85.12% of simulations. The probabilities of each strategy to be cost-effective against the variation of WTP threshold (0-150,000 USD/QALY) were shown in acceptability curves (Fig. 3). At a WTP threshold of 50,000 USD/QALY, the probability to be cost-effective was 85.06%, 14.86%, and 0.08% for IMQ + ID, IM, and ID, respectively.

## Discussion

The present study is the first analysis to evaluate the cost-effectiveness of ID administration of HBV Sci-B-Vac with pre-treatment IMQ cream in serologically negative dialysis patients from the US healthcare provider perspective. Our findings showed that the ID administration (with IMQ pre-treatment), with improved immune responses to HBV vaccination, resulted in the lowest incidences of hepatitis B-related events and thereby gained the highest QALYs. The ID needle and IMQ cream were considered as additional resource items in the IMQ + ID group versus the IM group. The ID needle syringe (USD10 per unit) and IMQ cream (USD3.36 per sachet) increased the cost of ID vaccination with IMQ pre-treatment by approximately 40% (USD13.36) when compared to IM vaccination (USD33.8 per dose). The cost reduction generated by lowering hepatitis B-related events in the IMQ + ID group therefore only partially offset the vaccination cost, and the IMQ + ID group was effective with higher QALY gain at increased costs (and ICER less than WTP) versus the IM group. When compared to the ID group, the additional cost of IMQ cream in the IMQ + ID group was fully offset by the cost avoidance generated by the reduced hepatitis B-related events.

A prospective randomized clinical trial examined the ID and IM re-vaccination in chronic dialysis patients (who were non-responsive to IM HBV vaccination) with follow-up period up to 20 months [46]. The results demonstrated significantly higher immunogenicity in the ID group (seroconversion rate 96% in ID group versus 40% in IM group; *p* = 0.0001) at one month after re-vaccination completion. Additionally, six months after vaccination completion, the geometric mean titers remained significantly higher in the ID group (26.5mUI/ml) than in the IM group (6.3mUI/ml) (*p* = 0.0001). The cost of the ID re-vaccination (USD 92) was lower than that of IM re-vaccination (USD 138), primarily due to the lower dose required in the ID group (20 µg × 4 doses) compared to the IM group (40 µg× 3 doses) [46]. Another prospective study evaluated the costs of a high-dose ID HBV vaccination strategy with a maintenance program in hemodialysis patient [47]. The ID HBV vaccination was given for 20 µg every 2 weeks until a titer of 20 IU/L was reached (maximum dose 240 µg) and the reinforced schedule was administered using a monthly single ID dose of 20 µg when anti-HBs titer declined below 20 IU/L. The mean cost of high-dose ID HBV vaccination was €127, lower than the cost of standard IM program (40 µg × 3 doses for both initial and maintenance phases) (€200.4) [47]. The cost-saving in ID (versus IM) vaccination was demonstrated in both studies, yet these studies did not consider the unit cost of ID needle springe or long-term health outcomes. Our study adopted a long-term model-based cost-effectiveness analysis to evaluate ID HBV vaccination strategies, incorporating QALYs and potential cost offsets from reduced hepatitis B-related complications. Our study also evaluated an HBV vaccination option using ID administration with IMQ pre-treatment to further enhance immune response, and the findings provided health economics evidence for optimizing HBV vaccination protocols in chronic dialysis patients.

Addressing the challenge of suboptimal vaccine responsiveness and durability is important for HBV vaccination in dialysis patients. Our model considered both vaccine responsiveness (upon completion of vaccination) and waning immunity over time in dialysis patients. The cost-effectiveness of the IMQ + ID group was mainly generated by the highest SPR and slowest decline in anti-HBs titers of all three groups. Our findings showed vaccination strategies that produce high initial response rates and sustained immunity are potentially cost-effective. The findings of the present study therefore support further clinical research with long-term follow-up to determine the duration of seroprotection achieved by IMQ + ID.

The study has some limitations. Due to the scarcity of clinical data on HBV vaccination and HBV infection in dialysis patients, some of the model inputs were extrapolated from the general population (including clinical inputs for proportion of symptomatic acute HBV infection, transition probability of inactive HBsAg carrier to seroprotected, CHB, compensated cirrhosis and HCC). It may therefore limit the generalizability of results to dialysis patients. Also, the long-term seroprotection durability of IMQ + ID in dialysis patients is yet to be investigated. The extrapolation of the proportion of anti-HBs-positive persons based on the initial anti-HBs titer levels might introduce some uncertainties to the model outcomes. The cost of ID needle springe was referenced from one online supplier, and other suppliers with competitive pricing were not fully identified. It may potentially overestimate the costs of ID vaccination. The healthcare service costs in this study were derived from fees, prices, DRG tariffs. These may not fully align with the economic cost estimates from the healthcare provider’s perspective.

## Conclusions

In conclusion, ID administration of HBV Sci-B-Vac with pre-treatment IMQ cream in serologically negative dialysis patients appears to reduce hepatitis B-related events and was cost-effective from the perspective of US healthcare providers.

## Supplementary Information

Below is the link to the electronic supplementary material.

Supplementary Material 1

## Data Availability

All data used as model inputs in the Markov model and data generated by the Markov model are provided in tables, figures and text of the manuscript and supplementary materials.

## Abbreviations

anti-HBs: Hepatitis B surface antibody
CHB: Chronic hepatitis B
CKD: Chronic kidney disease
DRGs: Diagnosis related groups
ESRD: End-stage renal disease
HBeAg: Hepatitis B e-antigen
HBsAg: Hepatitis B surface antigen
HBV: Hepatitis B virus
HCC: Hepatocellular carcinoma
ICER: Incremental cost-effectiveness ratio
ID: Intradermal
IM: Intramuscular
IMQ: Imiquimod
QALY: Quality-adjusted life-year
SPR: Seroprotection rate
TLR7: Toll-like receptor 7
USRDS: United States Renal Data System
WTP: Willingness-to-pay

## References

[1] Chu C-M. Natural history of chronic hepatitis B virus infection in adults with emphasis on the occurrence of cirrhosis and hepatocellular carcinoma. J Gastroenterol Hepatol. 2000;15(s2):E25–30. 10.1046/j.1440-1746.2000.02097.x. 10921378 10.1046/j.1440-1746.2000.02097.x

[2] Poh Z, Goh BB, Chang PE, Tan CK. Rates of cirrhosis and hepatocellular carcinoma in chronic hepatitis B and the role of surveillance: a 10-year follow-up of 673 patients. Eur J Gastroenterol Hepatol. 2015;27(6):638–43. 10.1097/meg.0000000000000341. 25831135 10.1097/MEG.0000000000000341 PMC4415961

[3] Lim JK, Nguyen MH, Kim WR, Gish R, Perumalswami P, Jacobson IM. Prevalence of chronic hepatitis B virus infection in the united States. Am J Gastroenterol. 2020;115(9):1429–38. 10.14309/ajg.0000000000000651. 32483003 10.14309/ajg.0000000000000651

[4] Cao YL, Wang SX, Zhu ZM. Hepatitis B viral infection in maintenance Hemodialysis patients: a three year follow-up. World J Gastroenterol. 2007;13(45):6037–40. 10.3748/wjg.v13.45.6037. 18023096 10.3748/wjg.v13.45.6037 PMC4250887

[5] Edey M, Barraclough K, Johnson DW. Review article: hepatitis B and Dialysis. Nephrology. 2010;15(2):137–45. 10.1111/j.1440-1797.2009.01268.x. 20470270 10.1111/j.1440-1797.2009.01268.x

[6] Fabrizi F, Martin P, Hepatitis B. Virus infection in Dialysis patients. Am J Nephrol. 2000;20(1):1–11. 10.1159/000013548. 10644861 10.1159/000013548

[7] Centers for Disease Control and Prevention. Hepatitis B vaccination: information for healthcare providers. [cited 2024 Apr 5]. Available from: https://www.cdc.gov/vaccines/vpd/hepb/hcp/index.html#recs.

[8] Buti M, Viladomiu L, Jardi R, Olmos A, Rodriguez JA, Bartolome J, et al. Long-Term immunogenicity and efficacy of hepatitis B vaccine in Hemodialysis patients. Am J Nephrol. 2008;12(3):144–7. 10.1159/000168436. 10.1159/000168436 1415374

[9] Mulley WR, Le STT, Ives KE. Primary seroresponses to double-dose compared with standard-dose hepatitis B vaccination in patients with chronic kidney disease: a systematic review and meta-analysis. Nephrol Dial Transpl. 2016;32(1):136–43. 10.1093/ndt/gfv443. 10.1093/ndt/gfv443 26763670

[10] Feng Y, Shi X, Shi J, Gao L, Liu G, Cheng Y, et al. Immunogenicity, antibody persistence, and safety of the 60 µg hepatitis B vaccine in Hemodialysis patients: a multicenter, randomized, double-blind, parallel-controlled trial. Expert Rev Vaccines. 2017;16(10):1045–52. 10.1080/14760584.2017.1367667. 28803502 10.1080/14760584.2017.1367667

[11] Chiew Tong NK, Beran J, Kee SA, Miguel JL, SanNchez C, Bayas JM, et al. Immunogenicity and safety of an adjuvanted hepatitis B vaccine in pre-hemodialysis and Hemodialysis patients. Kidney Int. 2005;68(5):2298–303. 10.1111/j.1523-1755.2005.00689.x. 16221232 10.1111/j.1523-1755.2005.00689.x

[12] Yousaf F, Gandham S, Galler M, Spinowitz B, Charytan C. Systematic review of the efficacy and safety of intradermal versus intramuscular hepatitis B vaccination in end-stage renal disease population unresponsive to primary vaccination series. Ren Fail. 2015;37(7):1080–8. 10.3109/0886022X.2015.1055698. 26258528

[13] Milich DR, Thornton GB, Neurath AR, Kent SB, Michel ML, Tiollais P, et al. Enhanced immunogenicity of the pre-S region of hepatitis B surface antigen. Science. 1985;228(4704):1195–9. 10.1126/science.2408336. 2408336 10.1126/science.2408336

[14] Hung IF-N, Yap DY-H, Yip TP-S, Zhang RR, To KK-W, Chan K-H, et al. A Double-blind, randomized phase 2 controlled trial of intradermal hepatitis B vaccination with a topical Toll-like receptor 7 agonist Imiquimod, in patients on Dialysis. Clin Infect Dis. 2020;73(2):e304–11. 10.1093/cid/ciaa804. 10.1093/cid/ciaa804 32556176

[15] Goodkin DA, Bragg-Gresham JL, Koenig KG, Wolfe RA, Akiba T, Andreucci VE, et al. Association of comorbid conditions and mortality in Hemodialysis patients in Europe, Japan, and the united states: the dialysis outcomes and practice patterns study (DOPPS). J Am Soc Nephrol. 2003;14(12):3270–7. 10.1097/01.Asn.0000100127.54107.57. PubMed PMID: 00001751-200312000-00028. 10.1097/01.asn.0000100127.54107.57 14638926

[16] Neild GH. Life expectancy with chronic kidney disease: an educational review. Pediatr Nephrol. 2017;32(2):243–8. 10.1007/s00467-016-3383-8. 27115888 10.1007/s00467-016-3383-8 PMC5203814

[17] Rosenthal EM, Hall EW, Rosenberg ES, Harris A, Nelson NP, Schillie S. Assessing the cost-utility of preferentially administering Heplisav-B vaccine to certain populations. Vaccine. 2020;38(51):8206–15. 10.1016/j.vaccine.2020.10.067. 33160756 10.1016/j.vaccine.2020.10.067

[18] Finelli L, Miller JT, Tokars JI, Alter MJ, Arduino MJ. National surveillance of dialysis-associated diseases in the united States, 2002. Semin Dial. 2005;18(1):52–61. 10.1111/j.1525-139X.2005.18108.x. 15663766 10.1111/j.1525-139X.2005.18108.x

[19] Yin J, Ji Z, Liang P, Wu Q, Cui F, Wang F, et al. The doses of 10µg should replace the doses of 5µg in newborn hepatitis B vaccination in china: A cost-effectiveness analysis. Vaccine. 2015;33(31):3731–8. 10.1016/j.vaccine.2015.05.082. 26057138 10.1016/j.vaccine.2015.05.082

[20] Kuan RK, Janssen R, Heyward W, Bennett S, Nordyke R. Cost-effectiveness of hepatitis B vaccination using HEPLISAV™ in selected adult populations compared to Engerix-B^®^ vaccine. Vaccine. 2013;31(37):4024–32. 10.1016/j.vaccine.2013.05.014. 23707166 10.1016/j.vaccine.2013.05.014

[21] Doi H, Yoshio S, Yoneyama K, Kawai H, Sakamoto Y, Shimagaki T, et al. Immune determinants in the acquisition and maintenance of antibody to hepatitis B surface antigen in adults after First-Time hepatitis B vaccination. Hepatol Commun. 2019;3(6):812–24. 10.1002/hep4.1357. 31168515 10.1002/hep4.1357 PMC6545872

[22] Hsu Y-S, Chien R-N, Yeh C-T, Sheen IS, Chiou H-Y, Chu C-M, et al. Long-term outcome after spontaneous hbeag seroconversion in patients with chronic hepatitis B. Hepatology. 2002;35(6):1522–7. 10.1053/jhep.2002.33638. 12029639 10.1053/jhep.2002.33638

[23] Artru F, Louvet A, Glowacki F, Bellati S, Frimat M, Gomis S, et al. The prognostic impact of cirrhosis on patients receiving maintenance haemodialysis. Aliment Pharmacol Ther. 2019;50(1):75–83. 10.1111/apt.15279. 31087566 10.1111/apt.15279

[24] Hwang J-C, Weng S-F, Weng R-H. High incidence of hepatocellular carcinoma in ESRD patients: caused by high hepatitis rate or ‘Uremia’? A Population-based study. Jpn J Clin Oncol. 2012;42(9):780–6. 10.1093/jjco/hys100. 22782961 10.1093/jjco/hys100

[25] Wyld M, Morton RL, Hayen A, Howard K, Webster AC. A systematic review and Meta-Analysis of Utility-Based quality of life in chronic kidney disease treatments. PLoS Med. 2012;9(9):e1001307. 10.1371/journal.pmed.1001307. 22984353 10.1371/journal.pmed.1001307 PMC3439392

[26] Levy AR, Kowdley KV, Iloeje U, Tafesse E, Mukherjee J, Gish R, et al. The impact of chronic hepatitis B on quality of life: A multinational study of utilities from infected and uninfected persons. Value Health. 2008;11(3):527–38. 10.1111/j.1524-4733.2007.00297.x. 18179664 10.1111/j.1524-4733.2007.00297.x

[27] Centers for Disease Control and Prevention. CDC vaccine price list, updated February 1, 2024. [cited 2024 Apr 5]. Available from: https://www.cdc.gov/vaccines/programs/vfc/awardees/vaccine-management/price-list/index.html.

[28] Aesthetic Mangement Partners. MicronJet600 | 100 ct per box. [cited 2024 Apr 5]. Available from: https://shop.aestheticmanagementpartners.com/micronjet600-100-ct-per-box/.

[29] PharmacyChecker.com LLC. Imiquimod cream prices. [cited 2024 Apr 5]. Available from: https://www.pharmacychecker.com/imiquimod+cream/?src=drug-suggest#prices.

[30] Centers for Medicare & Medicaid Services. Search the Physician Fee Schedule, 2023. [cited 2024 Apr 5]. Available from: https://www.cms.gov/medicare/physician-fee-schedule/search.

[31] Centers for Medicare & Medicaid Services. Medicare fee for service for Parts A & B, MEDPAR. [cited 2024 Apr 5]. Available from: https://www.cms.gov/data-research/statistics-trends-and-reports/medicare-fee-for-service-parts-a-b/medpar.

[32] Nguyen MH, Burak Ozbay A, Liou I, Meyer N, Gordon SC, Dusheiko G, et al. Healthcare resource utilization and costs by disease severity in an insured National sample of US patients with chronic hepatitis B. J Hepatol. 2019;70(1):24–32. 10.1016/j.jhep.2018.09.021. Epub 20181001. 30287341 10.1016/j.jhep.2018.09.021

[33] Pondé RAA. Expression and detection of anti-HBs antibodies after hepatitis B virus infection or vaccination in the context of protective immunity. Arch Virol. 2019;164(11):2645–58. 10.1007/s00705-019-04369-9. 31399876 10.1007/s00705-019-04369-9

[34] Sonnenberg FA, Beck JR. Markov models in medical decision making:a practical guide. Med Decis Mak. 1993;13(4):322–38. 10.1177/0272989x9301300409. 10.1177/0272989X9301300409 8246705

[35] Briggs A, Claxton K, Sculpher M. Decision modelling for health economic evaluation. Oxford University Press; 2006.

[36] Ribot S, Rothstein M, Goldblat M, Grasso M. Duration of hepatitis B surface antigenemia (HBs Ag) in Hemodialysis patients. Arch Intern Med. 1979;139(2):178–80. 10.1001/archinte.1979.03630390036015. 434972

[37] United States Renal Data System. Annual Data Report, End Stage Renal Disease, Chap. 6: Mortality. [cited 2024 Apr 5]. Available from: https://usrds-adr.niddk.nih.gov/2023/end-stage-renal-disease/6-mortality.

[38] Institute for Clinical and Economic Review. 2023 Value Assessment Framework. [cited 2025 Mar 10]. Available from: https://icer.org/wp-content/uploads/2023/09/ICER_2023_VAF_For-Publication_092523.pdf.

[39] Shemilt I, James T, Marcello M. A web-based tool for adjusting costs to a specific target currency and price year. Evid Policy. 2010;6(1):51–9. 10.1332/174426410x482999.

[40] U.S. Bureau of Labor Statistics. Consumer Price Index for All Urban Consumers: Medical Care in U.S. City Average [CPIMEDSL], retrieved from FRED, Federal Reserve Bank of St. Louis. [cited 2024 Apr 5]. Available from: https://fred.stlouisfed.org/series/CPIMEDSL.

[41] Drummond MF, Sculpher MJ, Claxton K, et al. Methods for the economic evaluation of health care programmes. 4th ed. Oxford: Oxford University Press; 2015.

[42] Neumann PJ, Kim DD. Cost-effectiveness thresholds used by study Authors, 1990–2021. JAMA. 2023;329(15):1312–4. 10.1001/jama.2023.1792. 37071104 10.1001/jama.2023.1792 PMC10114019

[43] Alastair M, Gray PM, Clarke. Jane L. Wolstenholme. Sarah Wordsworth. Applied methods of Cost-Effectiveness analysis in health care. Oxford: Oxford University Press; 2011. p. 253.

[44] Fenwick E, Claxton K, Sculpher M. Representing uncertainty: the role of cost-effectiveness acceptability curves. Health Econ. 2001;10(8):779–87. 10.1002/hec.635. 11747057 10.1002/hec.635

[45] Black WC. The CE plane: a graphic representation of cost-effectiveness. Med Decis Mak. 1990;10(3):212–4. 10.1177/0272989x9001000308. PubMed PMID: 2115096. 10.1177/0272989X9001000308 2115096

[46] Fabrizi F, Andrulli S, Bacchini G, Corti M, Locatelli F. Intradermal versus intramuscular hepatitis b re-vaccination in non-responsive chronic Dialysis patients: a prospective randomized study with cost-effectiveness evaluation. Nephrol Dial Transpl. 1997;12(6):1204–11. 10.1093/ndt/12.6.1204. 10.1093/ndt/12.6.1204 9198052

[47] Mat O, Mestrez F, Beauwens R, Muniz-Martinez M-C, Dhaene M. Primary high-dose intradermal hepatitis B vaccination in hemodialysis: Cost-effectiveness evaluation at 2 years. Hemodial Int. 2006;10(1):49–55. 10.1111/j.1542-4758.2006.01174.x. 16441827 10.1111/j.1542-4758.2006.01174.x

