# Supplementary material for: Intradermal hepatitis B vaccination with imiquimod pretreatment in dialysis patients: a cost-effectiveness analysis
Source: Cost Eff Resour Alloc. 2025 Dec 2;24:4. doi: 10.1186/s12962-025-00682-0 (PMC12777468; doi:10.1186/s12962-025-00682-0)
Supplement: Supplementary file 1 — Supplementary Material 1 [file 12962_2025_682_MOESM1_ESM.docx]

**Supplementary materials**

**Title: Intradermal Hepatitis B Vaccination with Imiquimod Pretreatment in Dialysis Patients: A Cost-effectiveness Analysis**

**Table S1.** Search strategy of MEDELINE

**Figure S1.** Flow diagram of literature search and selection process for clinical inputs

**Table S2.** Model inputs details in the sensitivity analysis

**Table S1.** Search strategy of MEDELINE

| # | Query |
| --- | --- |
| 1 | (renal dialysis patients or kidney failure or hemodialysis or peritoneal dialysis or hemodiafiltration).mp. |
| 2 | (hepatitis B or hepatitis B infection or chronic hepatitis B).mp. |
| 3 | (vital statistics or morbidity or mortality or incidence or prevalence or hospital* or general practitioner* or disease burden or natural history).mp. |
| 4 | 1 and 2 |
| 5 | 3 and 4 |
| 6 | (US or American or the United States).mp. |
| 7 | 5 and 6 |
| 8 | limit 7 to (English language and yr="2000 - 2024) |


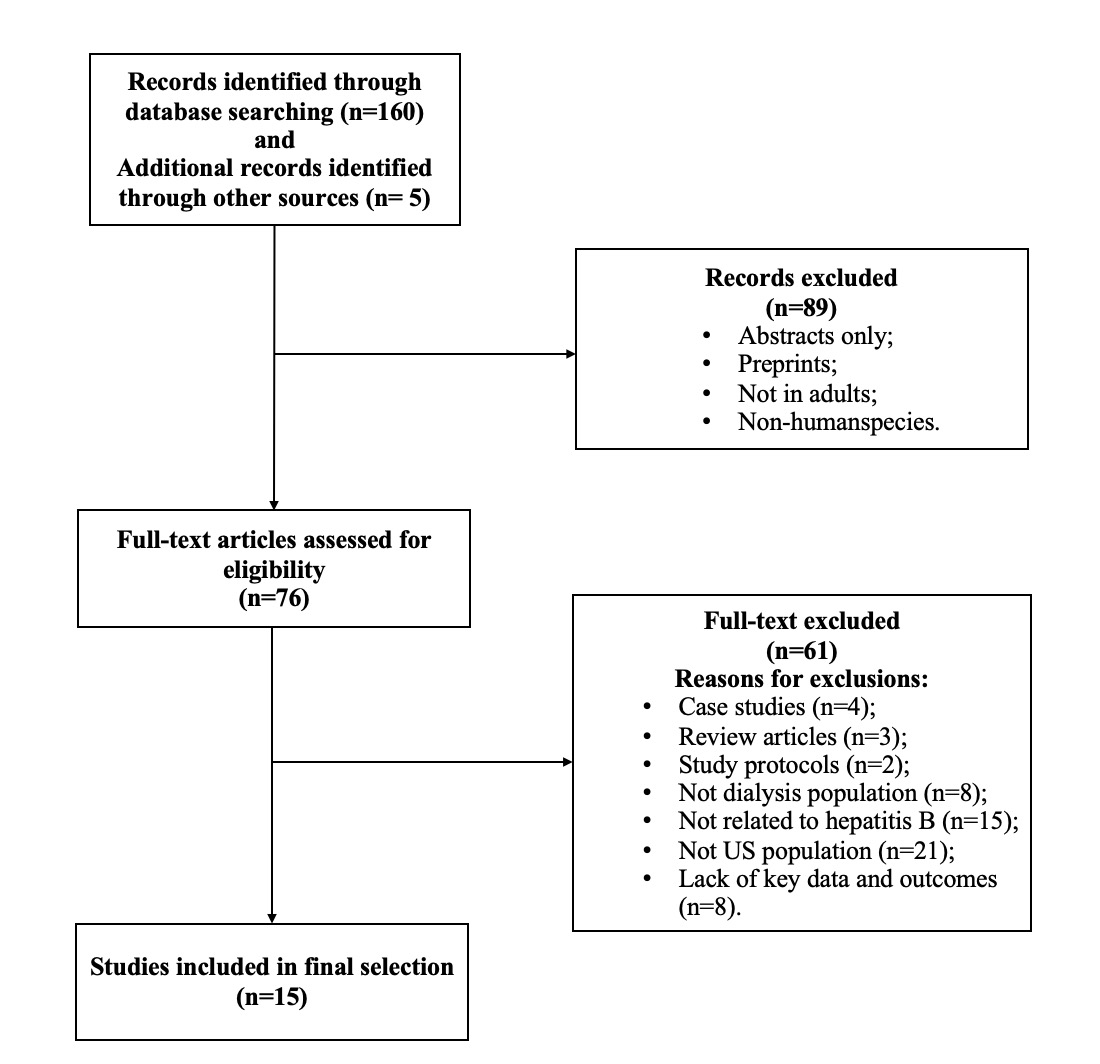


**Figure S1**. Flow diagram of literature search and selection process for clinical inputs

**Table S2** Model inputs details in the sensitivity analysis

| **Parameters** | **Base-case** | **Range for sensitivity analysis** | **Level of uncertainty^a^** | **Distribution** | **Alpha/Mean** | **Beta/SE** | **Reference** |
| --- | --- | --- | --- | --- | --- | --- | --- |
| **Clinical inputs** |  |  |  |  |  |  |  |
| Vaccination seroprotection rate |  |  |  |  |  |  | [1] |
| IMQ+ID | 0.9690 | 0.7750-1.0000 | 95% CIs | Beta | Alpha=31 | Beta=1 |  |
| ID | 0.7420 | 0.5940-0.8900 | 95% CIs | Beta | Alpha=23 | Beta=9 |  |
| IM | 0.4840 | 0.3870-0.5810 | 95% CIs | Beta | Alpha=15 | Beta=16 |  |
| Yearly incidence of acute HBV infection among unvaccinated dialysis patients | 0.0017 | 0.0013-0.0020 | ±20% | Beta | Alpha=69 | Beta=41305 | [2, 3] |
| Proportion of symptomatic acute HBV infection | 0.3000 | 0.1500-0.4500 | Upper/lower limits | Beta | Alpha=26 | Beta=163 | [4] |
| Proportion of fulminant hepatitis among acute HBV infection | 0.0830 | 0.0670-0.1000 | ±20% | Beta | Alpha=88 | Beta=968 | [5] |
| Transition probability in dialysis patients (yearly) |  |  |  |  |  |  |  |
| Seroprotected to Susceptible |  |  |  |  |  |  | [6] |
| IMQ+ID | 0.0480 | 0.0350-0.0660 | 95% CIs | Beta | Alpha=34 | Beta=679 |  |
| ID | 0.1280 | 0.1090-0.1500 | 95% CIs | Beta | Alpha=143 | Beta=972 |  |
| IM | 0.3430 | 0.2990-0.3920 | 95% CIs | Beta | Alpha=134 | Beta=256 |  |
| Acute HBV infection to CHB | 0.2725 | 0.2180-0.3270 | ±20% | Beta | Alpha=75 | Beta=38 | [5] |
| Fulminant hepatitis to CHB | 0.0710 | 0.0530-0.0890 | Upper/lower limits | Dirichlet | Alpha=71 | - | [2] |
| Fulminant hepatitis to Liver transplant | 0.0010 | 0.0007-0.0020 | Upper/lower limits | Dirichlet | Alpha=1 | - | [2] |
| Fulminant hepatitis to Death | 0.6700 | 0.5360-0.8040 | ±20% | Dirichlet | Alpha=670 | - | [2] |
| CHB to Inactive HBsAg carrier | 0.1110 | 0.0710-0.2290 | Upper/lower limits | Dirichlet | Alpha=111 | - | [2] |
| CHB to Compensated cirrhosis | 0.2000 | 0.1600-0.2400 | ±20% | Dirichlet | Alpha=200 | - | [5] |
| CHB to HCC | 0.2000 | 0.1600-0.2400 | ±20% | Dirichlet | Alpha=200 | - | [5] |
| Inactive HBsAg carrier to Seroprotected | 0.0054 | 0.0043-0.0065 | ±20% | Dirichlet | Alpha=5 | - | [7] |
| Inactive HBsAg carrier to CHB | 0.0449 | 0.0359-0.0538 | ±20% | Dirichlet | Alpha=45 | - | [7] |
| Inactive HBsAg carrier to Compensated cirrhosis | 0.0090 | 0.0072-0.0108 | ±20% | Dirichlet | Alpha=9 | - | [7] |
| Inactive HBsAg carrier to HCC | 0.0024 | 0.0019-0.0028 | ±20% | Dirichlet | Alpha=2 | - | [7] |
| Compensated cirrhosis to Decompensated cirrhosis | 0.1000 | 0.0800-0.1200 | ±20% | Dirichlet | Alpha=100 | - | [5] |
| Compensated cirrhosis to HCC | 0.0440 | 0.0352-0.0528 | ±20% | Dirichlet | Alpha=44 | - | [5] |
| Compensated cirrhosis to Death | 0.2326 | 0.1861-0.2791 | ±20% | Dirichlet | Alpha=233 | - | [8] |
| Decompensated cirrhosis to HCC | 0.0630 | 0.0300-0.0700 | Upper/lower limits | Beta | Alpha=36 | Beta=530 | [2] |
| Decompensated cirrhosis to Liver transplant | 0.0000 | 0.0000-0.0170 | Upper/lower limits | Gamma | Alpha=0.0006 | Beta=0.1600 | [2] |
| Decompensated cirrhosis to Death | 0.4090 | 0.3280-0.4910 | ±20% | Beta | Alpha=56 | Beta=81 | [8] |
| HCC to Liver transplant | 0.0000 | 0.0000-0.0170 | Upper/lower limits | Gamma | Alpha=0.0006 | Beta=0.1600 | [2] |
| HCC to Death | 0.5120 | 0.4100-0.6150 | ±20% | Beta | Alpha=46 | Beta=44 | [9] |
| Liver transplant to Death | 0.4800 | 0.3840-0.5760 | ±20% | Beta | Alpha=49 | Beta=54 | [5] |
| **Utility inputs** |  |  |  |  |  |  |  |
| Dialysis patients | 0.7000 | 0.6200-0.7800 | 95% CIs | Beta | Alpha=88 | Beta=38 | [10] |
| Hepatitis B-related state utility in general population |  |  |  |  |  |  |  |
| Symptomatic acute HBV infection | 0.7000 | 0.6300-0.7700 | Upper/lower limits | Beta | Alpha=88 | Beta=38 | [2] |
| Fulminant hepatitis | 0.3700 | 0.3300-0.4100 | Upper/lower limits | Beta | Alpha=242 | Beta=411 | [2] |
| Inactive HBsAg carrier | 0.8500 | 0.7700-0.9400 | Upper/lower limits | Beta | Alpha=57 | Beta=10 | [2] |
| CHB | 0.6700 | 0.6400-0.7000 | 95% CIs | Beta | Alpha=31 | Beta=15 | [11] |
| Compensated cirrhosis | 0.6600 | 0.6100-0.7100 | 95% CIs | Beta | Alpha=32 | Beta=16 | [11] |
| Decompensated cirrhosis | 0.3700 | 0.3200-0.4200 | 95% CIs | Beta | Alpha=60 | Beta=102 | [11] |
| HCC | 0.4300 | 0.3600-0.5000 | 95% CIs | Beta | Alpha=54 | Beta=72 | [11] |
| Liver transplant (year 1) | 0.5700 | 0.5400-0.6000 | 95% CIs | Beta | Alpha=41 | Beta=31 | [11] |
| Post Liver transplant | 0.6400 | 0.5900-0.6900 | 95% CIs | Beta | Alpha=34 | Beta=19 | [11] |
| **Cost inputs (USD)** |  |  |  |  |  |  |  |
| HBV Sci-B-Vac (per dose) | 34 | 27-41 | ±20% | Normal | Mean=33.80 | SE=3.45 | [12] |
| ID needle (per unit) | 10 | 8-12 | ±20% | Normal | Mean=10.00 | SE=1.53 | [13] |
| Imiquimod cream (per sachet) | 3 | 2-7 | Upper/lower limits | Normal | Mean=3.36 | SE=1.23 | [14] |
| Vaccination administration (first dose) | 20 | 18-27 | Upper/lower limits | Normal | Mean=20.33 | SE=2.27 | [15] |
| Vaccination administration (addition dose) | 15 | 13-19 | Upper/lower limits | Normal | Mean=14.57 | SE=1.43 | [15] |
| Treatment of symptomatic acute HBV infection | 10,239 | 3,594-16,883 | Upper/lower limits | Gamma | Alpha=9.12 | Beta=0.11 | [2] |
| Treatment of fulminant hepatitis | 28,026 | 22,421-33,631 | ±20% | Gamma | Alpha=96.03 | Beta=333.33 | [16] |
| Liver transplant surgery | 354,690 | 283,752-425,628 | ±20% | Gamma | Alpha=96.04 | Beta=3333.33 | [16] |
| State costs (annual) |  |  |  |  |  |  |  |
| Inactive HBsAg carrier | 183 | 146-220 | ±20% | Gamma | Alpha=96.04 | Beta=1.92 | [15] |
| CHB | 2,263 | 1,810-2,715 | ±20% | Gamma | Alpha=96.07 | Beta=23.81 | [17] |
| Compensated cirrhosis | 2,263 | 1,810-2,715 | ±20% | Gamma | Alpha=96.07 | Beta=23.81 | [17] |
| Decompensated cirrhosis | 19,970 | 15,976-23,964 | ±20% | Gamma | Alpha=96.04 | Beta=208.33 | [17] |
| HCC | 46,066 | 36,853-55,280 | ±20% | Gamma | Alpha=96.04 | Beta=500.00 | [17] |
| Liver transplant | 37,730 | 30,184-45,276 | ±20% | Gamma | Alpha=96.04 | Beta=400.00 | [17] |

HBV: hepatitis B virus; CHB: chronic hepatitis B; HCC: hepatocellular carcinoma; HBsAg: hepatitis B surface antigen; SE: Standard error

^a^The level of uncertainty was selected by upper and lower limits, 95% CIs, or plausible range using ±20% of base-case value (when both upper/lower range and 95% CI were lacking [18,19]).

**Reference**

1. Hung IF-N, Yap DY-H, Yip TP-S, Zhang RR, To KK-W, Chan K-H, et al. A Double-blind, Randomized Phase 2 Controlled Trial of Intradermal Hepatitis B Vaccination With a Topical Toll-like Receptor 7 Agonist Imiquimod, in Patients on Dialysis. Clin Infect Dis. 2020;73(2):e304-e11.

2. Rosenthal EM, Hall EW, Rosenberg ES, Harris A, Nelson NP, Schillie S. Assessing the cost-utility of preferentially administering Heplisav-B vaccine to certain populations. Vaccine. 2020;38(51):8206-15.

3. Finelli L, Miller JT, Tokars JI, Alter MJ, Arduino MJ. National surveillance of dialysis-associated diseases in the United States, 2002. Semin Dial. 2005;18(1):52-61.

4. Yin J, Ji Z, Liang P, Wu Q, Cui F, Wang F, et al. The doses of 10μg should replace the doses of 5μg in newborn hepatitis B vaccination in China: A cost-effectiveness analysis. Vaccine. 2015;33(31):3731-8.

5. Kuan RK, Janssen R, Heyward W, Bennett S, Nordyke R. Cost-effectiveness of hepatitis B vaccination using HEPLISAV™ in selected adult populations compared to Engerix-B® vaccine. Vaccine. 2013;31(37):4024-32.

6. Doi H, Yoshio S, Yoneyama K, Kawai H, Sakamoto Y, Shimagaki T, et al. Immune Determinants in the Acquisition and Maintenance of Antibody to Hepatitis B Surface Antigen in Adults After First-Time Hepatitis B Vaccination. Hepatol Commun. 2019;3(6):812-24.

7. Hsu Y-S, Chien R-N, Yeh C-T, Sheen IS, Chiou H-Y, Chu C-M, et al. Long-term outcome after spontaneous HBeAg seroconversion in patients with chronic hepatitis B. Hepatology. 2002;35(6):1522-7.

8. Artru F, Louvet A, Glowacki F, Bellati S, Frimat M, Gomis S, et al. The prognostic impact of cirrhosis on patients receiving maintenance haemodialysis. Aliment Pharmacol Ther. 2019;50(1):75-83.

9. Hwang J-C, Weng S-F, Weng R-H. High Incidence of Hepatocellular Carcinoma in ESRD Patients: Caused by High Hepatitis Rate or ‘Uremia’? A Population-based Study. Jpn J Clin Oncol. 2012;42(9):780-6.

10. Wyld M, Morton RL, Hayen A, Howard K, Webster AC. A Systematic Review and Meta-Analysis of Utility-Based Quality of Life in Chronic Kidney Disease Treatments. PLoS Med. 2012;9(9):e1001307.

11. Levy AR, Kowdley KV, Iloeje U, Tafesse E, Mukherjee J, Gish R, et al. The Impact of Chronic Hepatitis B on Quality of Life: A Multinational Study of Utilities from Infected and Uninfected Persons. Value Health. 2008;11(3):527-38.

12. Centers for Disease Control and Prevention. CDC Vaccine Price List, Updated February 1, 2024 [cited 2024 Apr 5]. Available from: <https://www.cdc.gov/vaccines/programs/vfc/awardees/vaccine-management/price-list/index.html>.

13. Aesthetic Mangement Partners. MicronJet600 | 100 ct per box [cited 2024 Apr 5]. Available from: <https://shop.aestheticmanagementpartners.com/micronjet600-100-ct-per-box/>.

14. PharmacyChecker.com LLC. Imiquimod cream Prices [cited 2024 Apr 5]. Available from: [https://www.pharmacychecker.com/imiquimod+cream/?src=drug-suggest#](https://www.pharmacychecker.com/imiquimod+cream/?src=drug-suggest)!

15. Centers for Medicare & Medicaid Services. Search the Physician Fee Schedule, 2023 [cited 2024 Apr 5]. Available from: <https://www.cms.gov/medicare/physician-fee-schedule/search>.

16. Centers for Medicare & Medicaid Services. Medicare fee for service for Parts A & B, MEDPAR [cited 2024 Apr 5]. Available from: <https://www.cms.gov/data-research/statistics-trends-and-reports/medicare-fee-for-service-parts-a-b/medpar>.

17. Nguyen MH, Burak Ozbay A, Liou I, Meyer N, Gordon SC, Dusheiko G, et al. Healthcare resource utilization and costs by disease severity in an insured national sample of US patients with chronic hepatitis B. J Hepatol. 2019;70(1):24-32. Epub 20181001.

18. Drummond MF, Sculpher MJ, Claxton K, et al. Methods for the economic evaluation of health care programmes. 4th ed. Oxford: Oxford University Press; 2015: 57-58.

19. Alastair M. Gray. Philip M. Clarke. Jane L. Wolstenholme. Sarah Wordsworth. Applied Methods of Cost-Effectiveness Analysis in Health Care. Oxford: Oxford University Press; 2011:253.
